# Supplementary material for: Ni-Catalyzed Enantioselective Intramolecular Mizoroki–Heck Reaction for the Synthesis of Phenanthridinone Derivatives
Source: J Org Chem. 2023 Jun 15;88(13):8203–26. doi: 10.1021/acs.joc.3c00202 (PMC10337041; doi:10.1021/acs.joc.3c00202)
Supplement: Supplementary file 2 — jo3c00202_si_002.zip [file jo3c00202_si_002.zip › Amides(N-H and N-Me)/S2e-I iPr-NH-ArI/1H iPr-NH-ArI S2e-I/2/pdata/1/email_DR-107-108 F21-22_2_1.pdf]

DR-107-108 F21-22
PROTON DMSO {D:\nmrusers\malachowski} BM 23

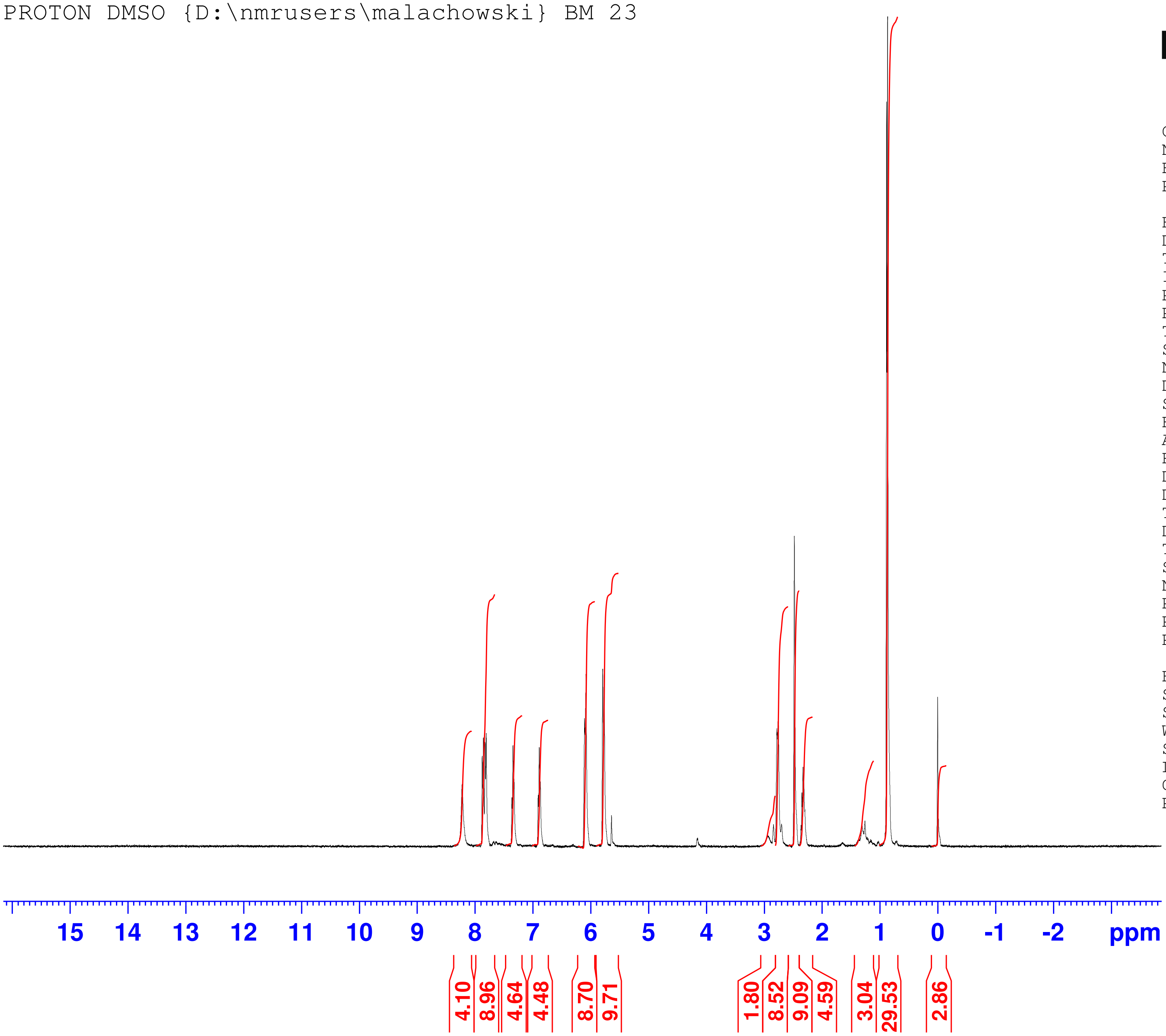

Current Data Parameters

NAME	DR-107-108 F21-22
EXPNO	2
PROCNO	1

F2 - Acquisition Parameters

Date_	20210222
Time	10.11 h
INSTRUM	spect
PROBHD	Z104450_0352 (
PULPROG	zg30
TD	65536
SOLVENT	DMSO
NS	16
DS	2
SWH	8012.820 Hz
FIDRES	0.244532 Hz
AQ	4.0894465 sec
RG	203
DW	62.400 usec
DE	17.30 usec
TE	373.1 K
D1	1.00000000 sec
TD0	1
SFO1	400.1524709 MHz
NUC1	1H
P0	4.25 usec
P1	12.75 usec
PLW1	12.14200020 W

F2 - Processing parameters

SI	65536
SF	400.1500122 MHz
WDW	EM
SSB	0
LB	0.30 Hz
GB	0
PC	1.00
